# Automatic volumetric estimates of the left and right atrium using dynamic PET

**DOI:** 10.1186/s13550-025-01352-1

**Published:** 2025-12-05

**Authors:** Hendrik J. Harms, Bent Roni Nielsen, Tanja Kero, Jonny Nordström, Stellan Mörner, Per Karlsson, Mark Lubberink, Henrik Wiggers, Lars P. Tolbod, Jens Sorensen

**Affiliations:** 1 Department of Surgical Sciences, Molecular Imaging and Physics, Uppsala University, Dag Hammarskjölds Väg 14B, plan 0, Uppsala, 75183 Sweden; 2 Clinical Institute, Aarhus University, Aarhus, Denmark; 3 MedTrace Pharma A/S, Hørsholm, Denmark; 4 Centre for Research and Development, Region Gävleborg/Uppsala University, Gävle, Sweden; 5 Department of Public Health and Clinical Medicine, Umeå University, Umeå, Sweden; 6 Department of Medical Sciences, Clinical Physiology, Uppsala University, Uppsala, Sweden

**Keywords:** Left atrium, Right atrium, O-15 water, Positron emission tomography, Echocardiography

## Abstract

**Background:**

Left (LAV) and right (RAV) atrial volumes are independent markers of cardiovascular risk in heart failure. Simultaneous assessment of myocardial blood flow (MBF) and atrial volumes might improve the clinical utility of cardiac PET. The aim of this study was to develop and validate an automated method for obtaining atrial volumes from dynamic myocardial perfusion PET scans without ECG-gating.

**Results:**

The atria were segmented automatically using first-pass data from [^15^O]-water PET at rest, combining voxel-wise images of bolus area-under-curve and arrival time. Data of multiple patient cohorts were analyzed: retrospective method development in 36 subjects with systolic heart failure with prospective validation in 59 subjects with same-day echocardiograms (primary hypertrophic cardiomyopathy (*n* = 25), suspected or known cardiac amyloidosis (*n* = 25) and healthy controls (*n* = 9)). Test-retest repeatability of PET was assessed in clinical chest pain patients scanned twice on the same day (*n* = 15). Segmentation was successful in all scans (n=125). PET and echocardiography correlated for LAV in the development cohort (r=0.83, p<0.001) and in the validation cohort (LAV: r=0.83, RAV: r=0.77, both p<0.001). In alignment with echocardiography, PET identified statistically significant differences between healthy controls and subjects with hypertrophied hearts for LAV index: 26 (interquartile range: 24-29) versus 41 (32-51) ml/m^2^, p<0.001, and RAV index: 31 (25-43) versus 48 (38-61) ml/m^2^, p=0.003). Test-retest reproducibility was excellent for LAV (intraclass correlation coefficient ICC=0.96, repeatability coefficient RPC=8.6 ml/m^2^) and for RAV (ICC=0.96, RPC=11.6 ml/m^2^).

****Conclusion**:**

Left and right atrial volumes can be extracted automatically, accurately and reproducibly using dynamic PET.

**Supplementary Information:**

The online version contains supplementary material available at 10.1186/s13550-025-01352-1.

## Introduction

Quantitative myocardial blood flow (MBF) measurements using Positron Emission Tomography (PET) are increasingly being used to assess ischemic burden in chronic coronary syndromes (CCS) [1–4] or to detect microvascular dysfunction (MVD) [5–7]. CCS and MVD often lead to cardiac dysfunction, increased filling pressures and heart failure (HF) [8]. As a consequence, structural and functional information is often required on top of MBF measurements to fully evaluate the disease severity [9–11]. Assessment of left ventricular (LV) structure and function is pivotal for treatment strategies and prognostication [12], and can to some extent be performed simultaneously with quantitative cardiac PET [9, 13, 14].

Increased left atrial volume (LAV) and right atrial volume (RAV) are considered indicative of elevated left ventricular filling pressures [15] and diastolic dysfunction, and has been associated with a higher incidence of cardiac events [15–20]. There is currently no automated approach available for measuring atrial volumes by PET.

We have shown in an animal model that differences in first-pass arrival time in various chambers can be measured accurately by dynamic PET [21], and that automated segmentation of both ventricles is feasible in humans [22]. When both sides of the heart are segmented, differences in arrival time between sub-regions could be used to segment the remaining cardiac chambers i.e. the atria. Therefore, by combining these findings, we have developed an algorithm that can automatically segment the LA and RA cavities. This approach enables us to assess atrial volumes using the standard PET protocols in clinical practice, without increasing protocol length. While anatomical methods (e.g. MRI, echocardiography) are the standard techniques for atrial volumetrics, adding atrial volumes increases protocol duration. Moreover, the potential ability of PET to simultaneously inform on both myocardial perfusion and aspects of structural heart disease could be relevant in case patient circumstances changed between echocardiography and PET.

The aim of this study is to validate this fully automated analysis of the first-pass phase of dynamic cardiac PET scans to obtain atrial volumes in addition to MBF.

## Methods

In this study, three patient cohorts were retrospectively defined. Subjects were included when they underwent an ^15^O-water PET in a clinical research study with a planned same-day echocardiography study as reference standard, or a second same-day ^15^O-water PET study for reproducibility.

### Patient population

The first cohort consisted of 36 subjects with documented systolic heart failure and LV ejection fraction (LVEF) below 45% (development cohort). This cohort was part of a sub-study of the LIVE study [23]. Group 2 (validation cohort) consisted of 25 subjects with documented high-risk primary hypertrophic cardiomyopathy (HCM), 25 subjects with known or suspected cardiac amyloidosis, and 9 healthy controls. HCM subjects were included as part of a clinical trial on predicting ventricular arrhythmias with PET [24], the other subjects were included as part of an ongoing research project in cardiac amyloidosis [25]. Finally, Group 3 (reproducibility cohort) consisted of 15 patients (mean age 68 ± 6 years, 13 male) with suspected angina, included in a previously reported test-retest PET study [26].

Patients in the development cohort were scanned at Aarhus University Hospital, Aarhus, Denmark, whilst patients in the validation and reproducibility cohorts were scanned at Uppsala University Hospital, Uppsala, Sweden. The research projects were approved by relevant ethical authorities and all subjects provided written informed consent.

## PET-CT

PET studies were conducted on either a Siemens Biograph TruePoint TrueV 64 PET/CT scanner (Group 1), a GE discovery MI (Group 2) or a GE Discovery ST (Group 3) with identical scanning protocols.

Following a low-dose attenuation CT, a 6-min list mode emission scan in the resting state was performed, starting simultaneously with bolus injection of 400 MBq of ^15^O-water as a 5–10 mL bolus (1 mL∙s^− 1^) in a peripheral vein, followed by a 35 mL saline flush (2.0 mL∙s^− 1^) as previously described [27]. To automate the injection and avoid bolus fractionation, the bolus was deposited in a saline-containing plastic tube, inserted between the venous catheter and an automated injection pump. The pump was programmed to operate at dual speeds. Emission data were acquired in list-mode and reconstructed in a dynamic series with 22 time frames (1 × 10 s, 8 × 5 s, 4 × 10 s, 2 × 15 s, 3 × 20 s, 2 × 30 s, 2 × 60 s) for ^15^O-water.

All dynamic images were reconstructed using the standard dynamic reconstruction protocols of each scanner, applying all routine corrections for attenuation, scatter, dead-time and decay as supplied by the vendor. In the reproducibility cohort, the patients left the scanner for at least 30 min after the first scan, and the second ^15^O-water scan included a new low-dose CT.

### Echocardiography

Echocardiographic data were obtained from the relevant study registries, involving four different observers. Several ultrasound systems (Vivid 7 or 9 (GE Healthcare)) were used. Images were analyzed off-line using commercially available software packages (echoPACS PC SW-only, GE Healthcare). LAV was measured using biplane area measurements at end-systole from 3 to 5 cardiac cycles, according to recommendations [28]. Right atrial area from an end-systolic four-chamber view was used as a substitute for RAV, as RA length was not routinely obtained in any of the cohorts. Right atrial area by echocardiography was not obtained in the cohort used for development.

### Calculation of atrial volume by PET

Right and left atrial volumes were obtained by tracking the arrival time and location of the first-pass bolus, and details can be found in [29]. This requires creation of images of both the bolus arrival time (t_mid_) and area under the curve (AUC). Both arrival time and AUC were, during segmentation, compared with t_mid_ and AUC of the LV and RV cavities, assuming an earlier bolus arrival in the atria than the ventricular cavities, as outlined below.

#### Parametric images of bolus area and arrival time

Data were analyzed using aQuant Research (MedTrace Pharma A/S, Hørsholm, Denmark) [30, 31]. As an initial step, the first-pass peak for each voxel in the dynamic data set was extracted automatically as described before [32]. The centroid time of this peak *t*_*mid*_, defined as

**Fig. 1:**
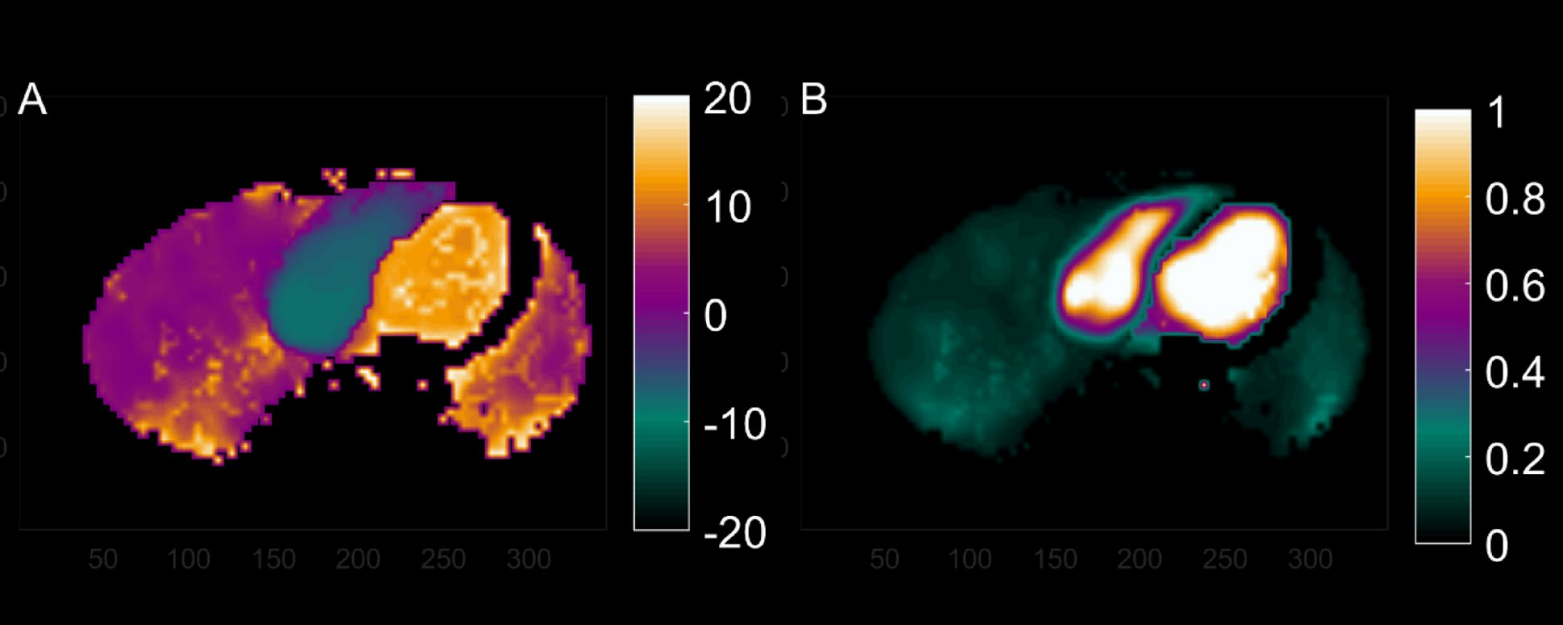
Typical example of an image of bolus arrival time t_mid_ (**A**), relative to that of the lungs, and area under curve AUC (**B**), normalized for the AUC of C_LV_(t). For display purposes, regions with bolus arrival time > 20 s after that of the left-ventricular cavity were removed from the images, effectively removing peripheral regions

$\:{t}_{mid}=\:\frac{\sum\:t\bullet\:C\left(t\right)}{\sum\:C\left(t\right)}$, and the area under the curve *AUC* were extracted. This results in a three-dimensional image for both *t*_*mid*_ (Fig. 1A) and for *AUC* (Fig. 1B), after which AUC was normalized such that the blood pool has a normalized area of 1. Regions with *AUC* greater than 2/3rd of that of the LV cavity, and short *t*_*mid*_ (less than 1.5 min) were identified and considered the blood region.

#### Segmentation of the left and right ventricle

Arterial and venous input functions were obtained by cluster analysis [31] using 6 clusters. After this, parametric images of MBF, Perfusable Tissue Fraction and the blood volume corrections V_A_ and V_RV_ were estimated using a basis function implementation of the single-tissue compartment model for cardiac ^15^O-water studies [31, 33]:

$$\begin{aligned}\:{C}_{T}\left(t\right)=\,&PTF\cdot MBF \cdot {C}_{A}\left(t\right)\:\otimes \:{e}^{-\frac{MBF}{{V}_{T}}\cdot\:t}\\&+{V}_{A}\cdot\:\:{C}_{A}\left(t\right)+\:{V}_{RV}\cdot\:\:{C}_{RV}\left(t\right)\:\end{aligned}$$

in which the distribution volume V_T_ was fixed to 0.91 ml∙g^−1^.

Resulting parametric images were automatically reoriented to short-axis images, and the left-ventricle was segmented using the methods described previously [30]. Using the same set of short-axis images, the right-ventricle was segmented in a similar way as the LV, except that V_RV_ instead of V_A_ images were used as starting point for the circumferential profiling as previously described [22].

LV and RV cavities were then defined as all regions inside of the myocardial regions at least 1.3 cm (twice an assumed PET resolution of 6.5 mm full-width at half maximum) away from segmented myocardial regions. For both cavities, the time-activity curve was obtained, the first-pass peak was extracted and resulting values for t_mid_ and AUC were stored as t_LV_, t_RV_, AUC_LV_ and AUC_RV_. AUC images were then normalized to AUC_LV_.

### Segmentation of the left and right atrium

Using parametric t_mid_ and AUC images, a blood mask was defined as all regions with normalized AUC > 2/3. Then the left- and right-heart were separated and cleaned. For the left heart, all regions with t_mid_ greater than t_LV_ plus the duration of two heartbeats were eliminated, as were all regions with t_mid_ less than that of the lungs. In addition, regions within the LV cavity were removed. This leaves a mask with a high blood volume and bolus arrival time approximately equal to that of the left atrium and aorta.

To separate the aorta from the left atrium, the LV outflow tract as defined in the segmentation of the LV was extrapolated in the atrial region until two distinct regions (LA and aorta) were obtained, of which the region with the lowest t_mid_ was kept as LA mask. The total volume of the remaining mask was obtained and used as estimate of mean LAV.

A similar approach was used for the right atrium. The superior vena cava was separated from the RA by assuming it to be a tube and the slices of the RA mask where a tube-like structure was found were removed from the final RA mask, the total volume of which was used as estimate of mean RAV.

### Data analysis

Correlation and agreement between PET and echocardiographic measures were assessed using linear regression and Bland Altman plots, including 95% limits of agreement and mean difference between measures. Two-way mixed, single score intra-class correlation coefficients (ICC) were used to assess reproducibility. Paired t-tests were used to assess the presence of systematic differences. Repeatability coefficient (RPC) in absolute numbers was defined as 2x the standard deviation (SD) of the difference, and Coefficient of Variance (CoV), a measure of relative repeatability (%), was calculated as (SD of differences/mean x 100). Differences between patient groups were assessed using non-parametric Kruskal-Wallis tests. There were many instances of skewed data distributions and grouped values are shown as median (interquartile range), unless otherwise stated. Matlab 2022B (Mathworks, Natick, MA) and JMP 18.1 (SAS Institute, Cary, NC) were used for statistical operations.

## Results

**Fig. 2:**
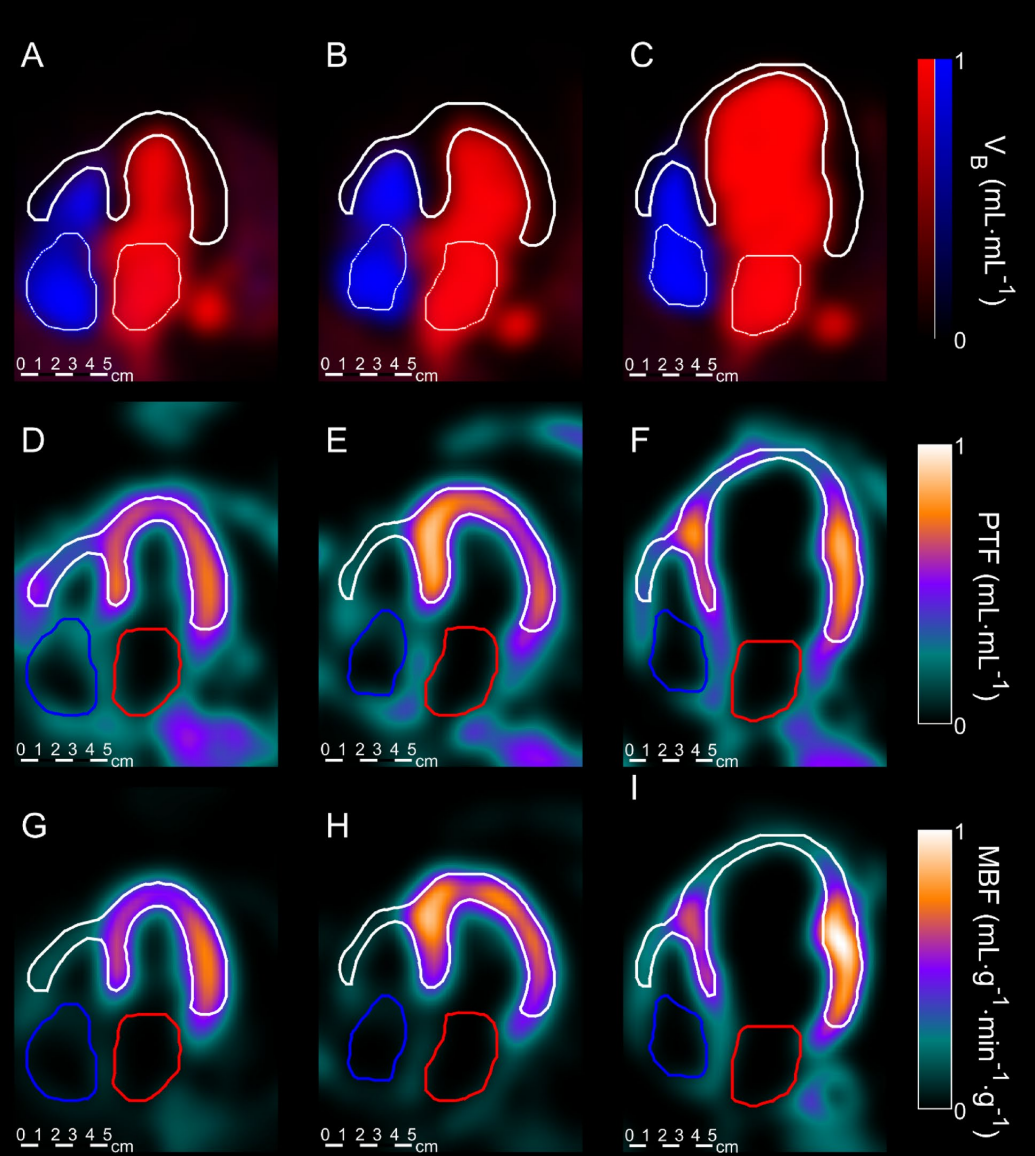
Typical example of venous and arterial blood volume images (**A**-**C**), PTF images (**D**-**F**) and MBF images (**G**-**I**) with segmented ventricles (white), left atria (red) and right atria (blue) indicated. (**A**, **D**, **G**) represent a healthy subject, (**B**, **E**, **H**) a HCM subject and (**C**, **F**, **I**) a HF subject. All images are displayed on the same physical scale.

**Fig. 3:**
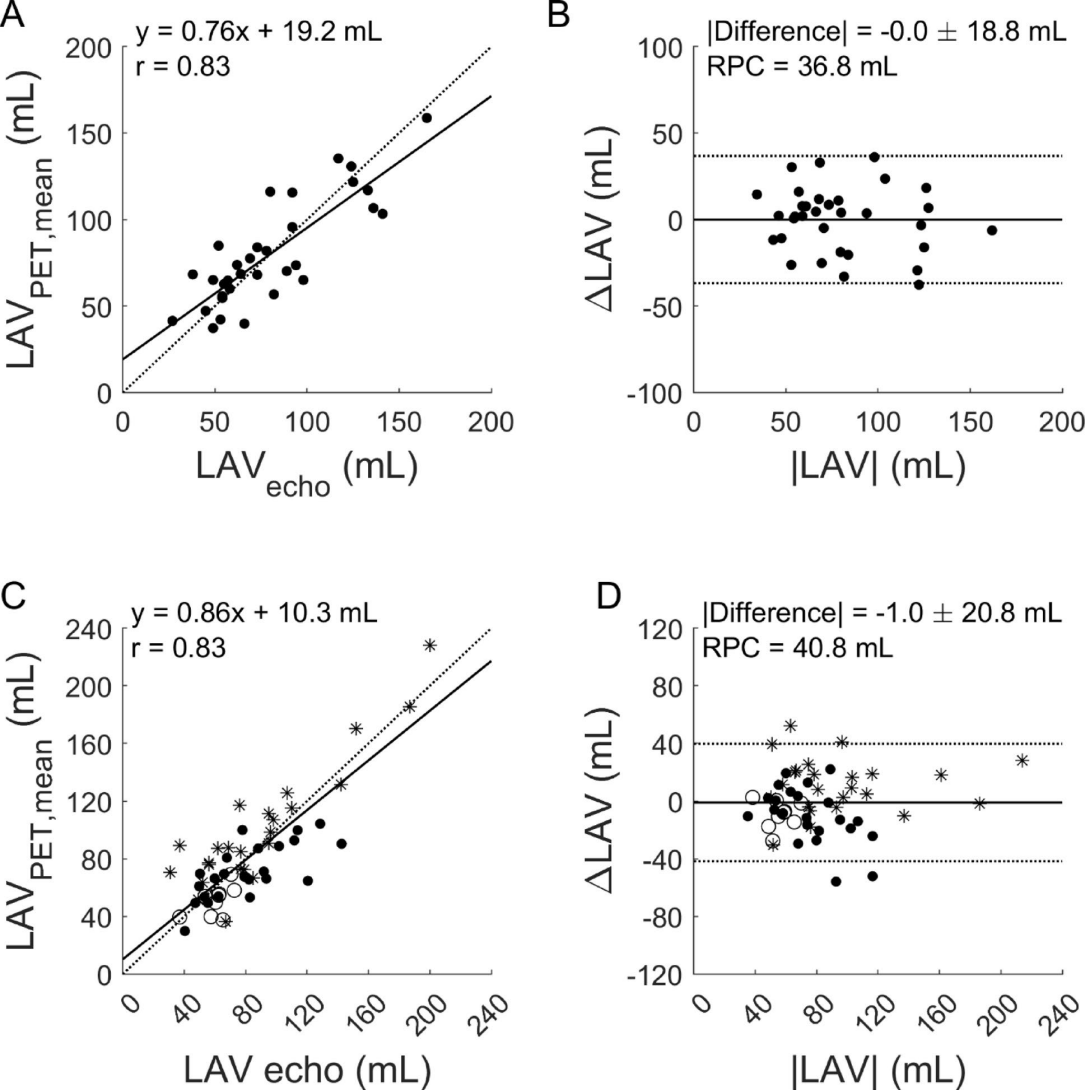
Correlation and Bland-Altman plots between maximal left atrial volume (LAV) based on echo and mean LAV based on PET in the development cohort (**A**-**B**) and the validation cohort (**C**-**D**, black dots: subjects with suspected or known cardiac amyloidosis; stars: hypertrophic cardiomyopathy; circles: healthy subjects).

Patient characteristics are shown in Table 1. Segmentation of the atria with PET was successful in all scans (*n* = 125), typical examples can be seen in Fig. 2 as a 4-chamber view. A significant correlation of PET- and echocardiography-derived LAV in the development cohort was found (*r* = 0.83, *p* < 0.001), shown in Fig. 3A-B. No systematic difference between PET and echo was found (*p* = 0.98), although slope and intercept of the linear fit were significantly different from 1 to 0, respectively (both *p* = 0.01).

**Table 1:** Patient characteristics and atrial volumes

	Cohorts					
	Development	Validation			Test-retest	
	HFrEF	HV	HCM	CA	CCS test	CCS retest
N	36	9	25	25	15	15
Sex (female/male)	2/34	5/4	6/19	5/20	2/13	-
Age (years)	66 (63–71)	56 (49–62)	57 (47–67)	70 (67–75)	67 (63–71)	-
BMI (kg/m2)	26 (23.8–29.4)	26.0 (20.2–27.6)	26.2 (24.0–30.2.0.2)	24.0 (20.2–25.2)	24.9 (24.1–28.7)	-
BSA	2.02 (1.89–2.11)	1.98 (1.66–2.07)	2.01 (1.83–2.12)	1.87 (1.77–1.95)	2.00 (1.87–2.11)	-
LVEF (%)	35 (29–41)	66 (59–69)	60 (55–65)	52 (45–66)	-	-
Atrial fibrillation (yes/no)	6/30	0/9	7/18	5/20	0/15	-
Echo LAV index (ml/m^2^)	35 (28–53)	34 (28–36)	38 (30–54)	40 (31–52)	-	-
Echo RA area index (cm^2^/m^2^))	-	8 (7–10)	9 (7–10)	11 (7–13)	-	-
PET LAV index (ml/m^2^)	35 (30–49)	26 (24–29)	44 (39–54)	37 (29–44)	28 (21–34)	32 (24–45)
PET RAV index (ml/m^2^)	41 (30–49)	30 (24–43)	43 (37–60)	53 (34–70)	44 (32–51)	43 (27–45)

HFrEF: heart failure with reduced ejection fraction. HV: healthy volunteers, HCM: hypertrophic cardiomyopathy. CA: suspected or known cardiac amyloidosis. CCS: chronic coronary syndrome. Echo: echocardiography. LAV: left atrial volume. RA: right atrial. Continuous variables are presented as median (interquartile range)

In the validation cohort, the correlation between LA volume based on PET and echocardiography was *r* = 0.98 with no significant difference between the two estimates (*p* = 0.073). Slope and intercept were significantly different from 1 to 0, respectively (*p* = 0.002 and *p* < 0.001). One subject had an extremely large LA (approximately 500 ml for both echocardiography and PET, see supplemental figure S1 online) - excluding this patient, the correlation remained significant (*r* = 0.83) with an overestimation of PET versus echocardiography (*p* = 0.010). However, slope and intercept were not significantly different from 1 to 0 (see Fig. 3C-D). A statistically significant correlation was found between RA volumes from PET and RA area derived from echo (*r* = 0.77, *p* < 0.001, Fig. 3).

**Fig. 4:**
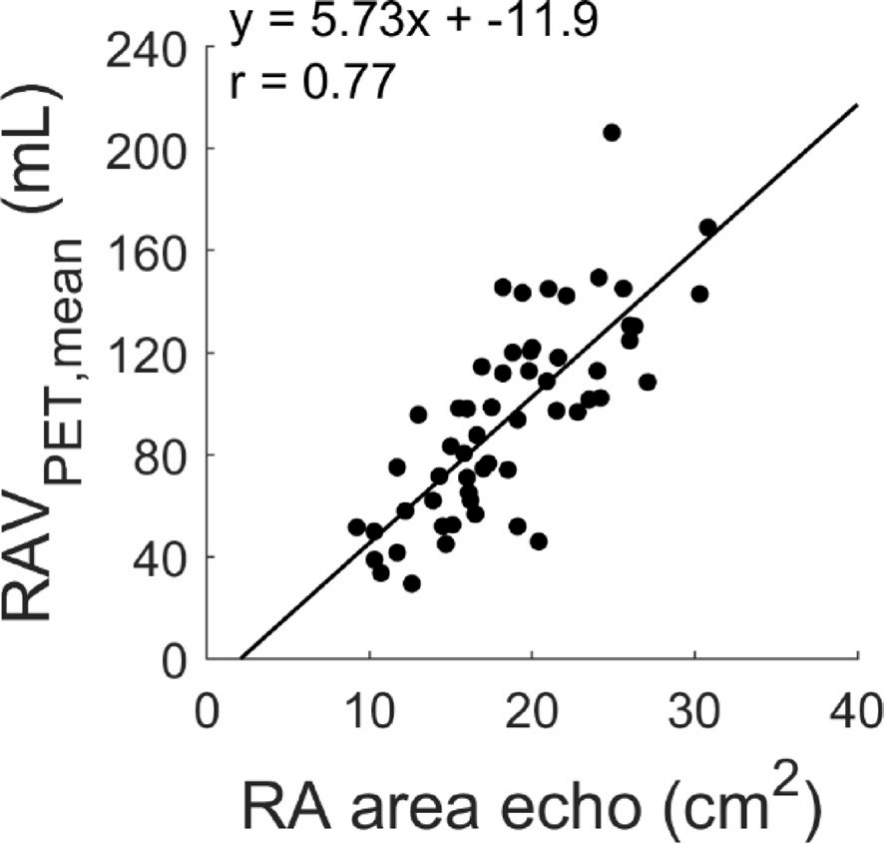
Correlation between RA area based on echocardiography (echo) and mean RAV based on PET in the prospective validation cohort

Finally, dual-scan test-retest reproducibility of the PET method was excellent with ICC = 0.93, RPC = 8.6 mL/m^2^ and CoV 9.9% (Fig. 4) for LAVI, and ICC = 0.96, RPC = 11.6 mL/m^2^ and CoV 18.7% for RAVI. PET observer reproducibility was high with an ICC of 0.99 and RPC of 5.6 mL/m^2^, and an ICC of 0.97 and RPC of 8.3 mL/m^2^ for LA and RA, respectively, when including all subjects. Observer variability for echocardiography was evaluated in the HCM subjects from the validation cohort, showing an ICC of 0.96 and RPC of 24.5 mL/m^2^ for LA and an ICC of 0.72 and RPC of 6.1 cm^2^ for RA area.

The median estimates for atrial volumes indexed by body surface area are shown in Table 1. LAV index in healthy volunteers was slightly higher for echocardiography than for PET (*p* = 0.031). PET identified statistically significant differences between healthy controls and subjects with hypertrophic cardiomyopathies for LAV index: 26 (interquartile range 24–29) versus 41 (32–51) ml/m^2^, *p* < 0.001, and for RAV index: 31 (25–43) versus 48 (38–61) ml/m^2^, *p* = 0.003).

**Fig. 5:**
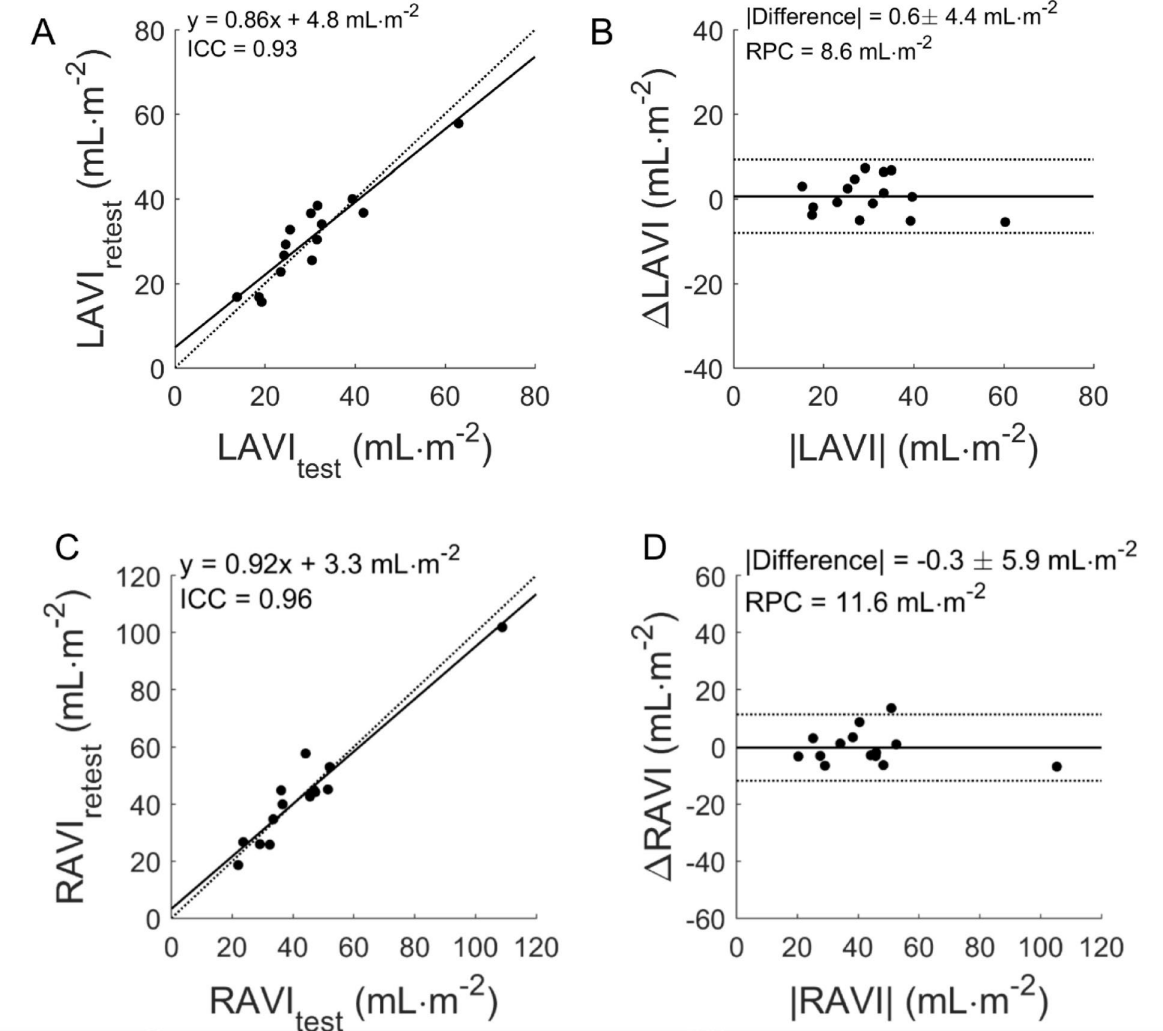
Correlation (**A**,**C**) and Bland Altman (**B**,**D**) plot of test-retest reproducibility of PET-based left atrial volume index (LAVI) (**A**,**B**) and right atrial volume index (RAVI) (**C**,**D**). Black and gray lines indicate the line of identity and the linear fit in (**A**,**C**) and the mean difference and the limits of agreement in (**B**,**D**). RPC: repeatability coefficient.

## Discussion

This study presents an automated method of segmenting the left and right atrium in dynamic ^15^O-water PET studies, yielding atrial volume information in addition to standard information MBF. A statistically significant correlation was found with the clinical gold standard of echocardiography and the method was highly reproducible in a test-retest setting, as well as in terms of observer reproducibility. The method only requires minor user input related to quality control of automated chamber segmentations, which is a routine part of quantifying MBF.

Combining classical indicator dilution curve analysis and kinetic modeling in dynamic PET imaging yields a wealth of spatial and physiologic information of cardiovascular interest. Voxel-wise application of first-pass curve analysis visualizes for example the time-point when half of the bolus has transited (t_mid_) and relative water volumes (AUC), as in Fig. 1. The kinetic model for water transport used with cardiac PET separates the quantitative parameters perfusable tissue fraction (PTF), myocardial blood flow (MBF, arterial blood (VA) and venous blood (VRV). These parameters can be visualized separately and in fused displays, as in Fig. 2. Combining the location of the ventricles with our earlier finding that bolus arrival times differ enough between the cardiac chambers and large vessels [21] to detect relevant time differences, we developed a method that can segment the left and right atria and reliably indicate their volumes using data of a standard MBF scan. Even though time differences are less than the shortest frame duration (5s), the method was found to be reliable, reproducible between scans and between observers. The degree of reproducibility does not appear to be inferior to echocardiography. We did not perform repeated echocardiography, but the 10% CoV for PET-LAV compares well to recent repeatability studies using echocardiography [34] and cardiac magnetic resonance imaging [35]. It should be noted that this study used 2D-echocardiography, which, while widely used, often underestimates LAV. 3D-echocardiography would be a better standard of truth, but no 3D-echocardiography data were available for the current study. A final validation in comparing LAV derived using the current PET-based method with those of 3D-echocardiography or cardiac MRI is warranted.

CoV for PET-RAV was poorer than for PET-LAV. Repeatability studies of RA volumes in literature are scarce. PET-based LAV and RAV estimates correctly showed significantly enlarged volumes in cardiomyopathy patients, compared to healthy controls, supporting the validity of the new method.

One previous study presented a PET-based approach to obtain LAV [36] using the first-pass of the injected bolus, similar to the current study. The method was based on ^82^Rb data with manual delineation of the early phase of the PET study projected on the attenuation CT images. Correlation versus echocardiography in that study was *r* = 0.70, which is in line or slightly lower than the correlations found in the current study. This suggests that first-pass images of PET can reliably be used to segment the LA in both a manual and automated fashion. To date, and to the best of our knowledge, no such studies exist for the RA. In addition, we did not attempt to estimate the prognostic relevance of atrial volumes in the current study, as we used small cohorts with very disparate pathologies, and therefore studies confirming the results of Koh et al. [36], as well as studies showing whether RAV has prognostic value over MFR and LAV, are encouraged.

Same-day PET and echocardiography in our small cohort of healthy controls showed a small but significant difference in LAV. Atrial volumes presented in this study represents average volumes based on non-ECG-gated PET images and no information regarding minimal and maximal atrial volume or emptying fraction is obtained. Care should be taken when comparing PET values with those obtained with other imaging modalities that do yield this information. However, average LAV by itself may be a marker of chronic LV pressure overload and was associated with prognosis [36]. Therefore, LAV obtained using the methods presented in this study may be beneficial in routinely identifying patients at risk when echocardiography was not available, irrespective of their perfusion or perfusion reserve.

PET data were acquired with three different scanners. Equal time-frame lengths for PET were used in all the sub-cohorts and were found to be adequate for the three scanner types available. The 5-second time resolution of early frames performed surprisingly well with indicator dilution approaches. Lowering the time resolution of early frames below the current 5 s using newer high-sensitivity scanners is not required for the tracer kinetic models applied for measuring myocardial perfusion but will likely increase the accuracy and utility of first-pass transit time measurements from PET [21].

## Limitations

The main limitation in this proof-of-principle study was the retrospective study design, combining the data of several different patient categories scanned on several different scanners. Echocardiography was performed and analyzed by several different observers, which might distort the statistics, and not all subjects underwent right atrial reference measurements. None the less, the results indicate that PET might be used for evaluation of bi-atrial enlargement. The small number of healthy controls included is not enough to state a PET-based cut-off for atrial enlargement, which is an aim of future studies.

RAV could only be compared with a 2-dimensional RA area estimate on echocardiography. Measuring RAV by area/length approaches was not routine practice with echocardiography when the original research studies were conducted, and these were the only data available that could serve as validation. A comparison of PET-RAV with a true 3-dimensional estimate of RAV with cardiac MRI or 3D echocardiography should be performed.

## Conclusion

Left and right atrial volumes can routinely be assessed using dynamic PET and analysis of the first-pass of the bolus through the heart. The methods presented in this study are fully automated and in theory independent of the tracer being used, enabling a routine and fully simultaneous assessment of both atrial volumes and myocardial blood flow

## Supplementary Information

Supplementary Material 1

## Data Availability

The datasets used and analysed during the current study are not publicly available due to GDPR restrictions but are available from the corresponding author on reasonable request.
