# Supplementary material for: Automatic volumetric estimates of the left and right atrium using dynamic PET
Source: EJNMMI Res. 2025 Dec 5;16:7. doi: 10.1186/s13550-025-01352-1 (PMC12796069; doi:10.1186/s13550-025-01352-1)
Supplement: Supplementary file 1 — Supplementary Material 1 [file 13550_2025_1352_MOESM1_ESM.docx]

## Supplemental file. Harms et al. **Automatic volumetric estimates of the left and right atrium using dynamic PET**


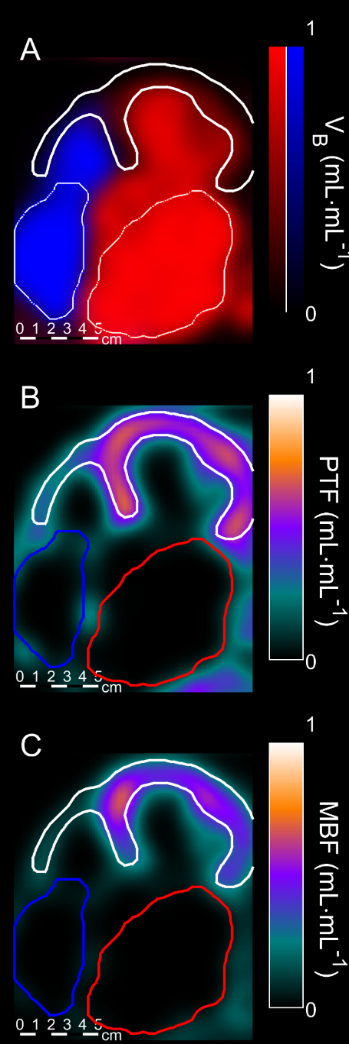


Supplemental figure 1S. PET analysis from one outlier among the cardiomyopathic subjects. Blood volume image (A), PTF image (B) and MBF image (C) with segmented ventricles (white), left atria (red) and right atria (blue) indicated. Left atrial volume was 480 mL by echocardiography and 460 mL by PET.
